# Lewy bodies and neuronal loss in subcortical areas and disability in non-demented older people: a population based neuropathological cohort study

**DOI:** 10.1186/1471-2318-9-22

**Published:** 2009-06-15

**Authors:** M Byford, C Brayne, I McKeith, M Chatfield, PG Ince, FE Matthews, MRC CFAS Neuropathology group

**Affiliations:** 1 Department of Public Health and Primary Care, University of Cambridge, Cambridge, UK; 2 Institute for Ageing and Health, Campus for Ageing and Vitality, Newcastle University, Newcastle upon Tyne UK; 3 Academic Unit of Pathology, University of Sheffield Medical School, Sheffield UK; 4 MRC Biostatistics Unit, Cambridge, UK

## Abstract

**Background:**

Functional disability, the loss of ability to carry out daily tasks unaided, is a major adverse outcome more common with increasing age. The potential contribution of neuropathological changes in subcortical areas of the brain associated with normal ageing may be a contributing factor to this loss of function. This study investigates the clinicopathological relationship between functional ability during life and pathological correlates identified at post mortem in an UK population of older people (66–102 years).

The aim is to examine the clinicopathological correlates of functional disability in subcortical neuronal populations of non-demented elderly individuals.

**Methods:**

156 non-demented participants in the brain donation programme of the Medical Research Council Cognitive Function and Ageing Study (MRC-CFAS) were included in this study. Neuropathological examination was based on the CERAD protocol; pathologies of interest were amyloid plaques, neurofibrillary tangles, Lewy bodies, vascular disease and neuronal loss. Self-reported functional ability was scored according to a combined activities of daily living and instrumental activities of daily living scale.

**Results:**

Functional disability was equally common in men and women over 65 years, and in both sexes disability was more common at older ages. Neuronal loss in several subcortical regions elevated the risk of functional disability by three-fold (95% CI 1.3–6.6). There was evidence for a relationship between Lewy bodies in the SN and functional disability.

**Conclusion:**

Neuronal loss in subcortical regions is associated with functional disability in the older population. The causal relationships are not defined and require further investigation.

## Background

Functional disability may be defined as 'the loss of ability to carry out daily tasks unaided'[1]. It has been estimated that 11% of men and 19% of women over the age of 65 years living in the UK are functionally disabled[2]. The effects of disease and ageing on an individual's functional ability, and in particular their performance of activities of daily living, is one of the most common problems that has an impact on the health and quality of life in older people. A priori assumptions about the cause of functional disability in the absence of an established diagnosed disease could suggest the contribution of a subclinical burden of morbidities associated with physical dysfunction (e.g. arthritis, cardiorespiratory disease, cerebrovascular disease). However, neurodegeneration associated with ageing, most especially Parkinson's disease (PD), is a cause of impaired physical function. The brain regions involved in PD are also vulnerable to other degenerative processes and can show neuronal loss in the absence of neuronal lesions. We have therefore analysed data from a large prospective, population-based cohort to investigate potential relationships between functional disability and cerebral degenerative disease in key subcortical regions (medulla, pons, midbrain and basal forebrain).

The worldwide increase in longevity, and the concomitant rise in functional disability, demands an understanding of how brain function in ageing, and the neuropathological substrates that underlie age-related decline, impact on physical function. A number of neuropathological alterations have been observed in the brains of older people compared to those dying at younger ages. The occurrence of lesions characteristic of Alzheimer' s disease, neurofibrillary tangles (NFT) and amyloid plaques, is an underappreciated feature of the brain in cognitively intact elderly individuals[3]. Several studies have shown that a substantial burden of Alzheimer-type pathology is found in aged individuals with normal cognitive function [4-9]. Pathological changes associated with Parkinson's disease, most notably the formation of Lewy bodies in the midbrain substantia nigra (SN), have also been reported in cognitively normal aged individuals. In Parkinson's disease, these lesions are associated with impaired gait initiation, limb rigidity and postural instability[10]. Like Alzheimer's pathology, the prevalence of Lewy bodies has been shown to increase with age[11]. In addition to NFT and plaques, neuronal loss is a characteristic feature of Alzheimer's disease and other neurodegenerative disorders. Neuronal loss may be associated with normal ageing, though research using different procedures for counting neurons suggests this may have been overestimated[12]. Vascular disease is a known cause of dementia, but also causes physical disability, e.g. through stroke. Recently there is also increasing interest in the role of small vessel disease as a substrate for functional decline.

Clinicopathological studies are usually based on comparisons of clinically defined case series, such as Alzheimer's disease [13] or stroke[14], and often include a relatively healthy, control brain comparison group using criteria that include restricted neuropathological burden. Few have explored the relationship between neuropathology and gait, movement disorder or functional disability in an unbiased sample of the ageing population among whom a sub-clinical threshold of such symptoms is common[15]. Associations between functional disability and pathologies typical of Alzheimer's disease (plaques and NFT), Lewy bodies[16] and neuronal loss in subcortical areas have been reported in small, mainly retrospective studies based on highly selected individuals[17,18]. It is important to see whether such findings extend to population-based cohorts[19], so that their relevance to the marked increase of functional disability in older age can be estimated. There has been some indication from imaging studies that vascular changes in subcortical regions are associated with a decline in physical ability [20,21].

In this paper we compare functional disability and subcortical neuropathology in brain donations to the Medical Research Council Cognitive Functioning and Ageing Study (MRC-CFAS), a prospective, longitudinal, population-based study of dementia and physical frailty that includes a brain donation programme. We test whether loss of functional disability is associated with Alzheimer-type pathology, Lewy bodies, vascular disease, and neuronal loss across subcortical areas.

## Methods

MRC-CFAS is a population based longitudinal study of the population aged 65 and older, including institutional settings with a brain donation programme. This study has been described fully elsewhere[4,22].

In summary, at each of five centres in England and Wales (Cambridgeshire, Gwynedd, Newcastle upon Tyne, Nottingham and Oxford), about 2,500 people agreed to a structured initial interview, which collected sociodemographic information, cognitive measures (including MMSE), medication and questions on activities of daily living (ADL) and instrumental activities of daily living (IADL).

A stratified subsample comprising 20% of those screened was selected for assessment, comprising the Geriatric Mental State (GMS version B3). This was repeated 2 years later with a re-screen plus a new 20% subsample of those not previously assessed together with a second assessment interview for those who had previously been assessed. Both assessment groups were followed up again six years after the first interview. The GMS provides an algorithmic dementia diagnosis.

Individuals selected for assessment were approached by a trained liaison officer to discuss brain donation and were asked to consider making a 'Declaration of Intent' to make a post-mortem brain donation. Donations relied on notification of the death of a donor to the study team, after which the next of kin were approached to give consent for brain donation and retention. The methods for post-mortem brain donation followed a standardised protocol[4]. The cerebral hemispheres were separated and sectioned in the coronal plane. One hemisphere was dissected, macroscopically examined and snap- frozen. The other half of the brain was formalin fixed for conventional paraffin embedding and histology [5].

This analysis is based on the first 355 brains donated from the five centres. We report clinicopathological associations of functional disability in the non-demented subsample of 155 donors for whom all data were available.

### Measures

#### Neuropathology assessment

Neuropathology was assessed following the Consortium to Establish a Registry of Alzheimer's Disease (CERAD) neuropathology protocol[23] based on semiquantitative assessment of the severity of specific lesions, including NFT, plaques, Lewy bodies and neuronal loss. NFT were assessed using a silver stain or immunocytochemistry to hyperphosphorylated tau. Amyloid plaques were assessed using silver staining or immunocytochemistry for β amyloid. LB were assessed by H&E staining or ubiquitin immunocytochemistry. The severity of the pathologies was scored as either none, mild, moderate or severe in five subcortical brain regions: SN, nucleus basalis of Meynert (NbM), raphé nuclei, locus ceruleus and dorsal efferent nucleus of vagus nerve (DEX). Apart from the SN none of these regions a regarded as predominantly associated with motor function. For each individual a global measure of severity was derived from the worst score across the five regions. Vascular disease was assessed in conventionally stained tissue sections. Lesions considered to be of potential significance included lacunes, severe arteriolar sclerosis, haemorrhage and marked expansion of perivascular spaces. We combined information on the presence of such lesions in each of the five subcortical regions. The neuropathology variables were recoded to a dichotomous variable (present and absent) for this analysis. Neuropathological examination was carried out blind to clinical or interview data. To ensure consistency between centres, inter-rater reliability was confirmed at the start of the study by circulation of microscopic slides. In this exercise, fewer than 5% of the scores assigned were more than one severity grade different among participants[24].

Missing information on the CERAD form was divided according to its nature. "Don't know" and "Not Available/Applicable" were assigned separate codes, but were recoded to zero (absent) for the analyses presented.

#### Functional disability

Information on self-reported functional disability was collected at all screening and assessment interviews. Questionnaires included questions on basic ADL, such as bathing, dressing and using the toilet, and IADL which include the more complex activities that are required for the maintenance of independence and optimum levels of functioning (e.g. shopping, cooking)[25]. From these data, a hierarchical functional disability status was derived[26]. ADL-IADL scores were grouped into mild (IADL only) and severe (IADL and/or ADL); both these levels indicate that individuals would require help at least several times per week (table 1).

**Table 1:** Questions used in the screen and combined screen and assessment interview to collect information on ADL and IADL.

**Question from screen/combined screen and assessment interview**	**Answers**
**ADL-IADL disability requiring help at least several times/week**	
• Are you able to wash all over or bath? • Are you able to prepare and cook a hot meal? (IADL) • Are you able to put on your shoes and socks or stockings?	• No, needs help • Yes with some difficulty • Yes with no difficulty
• Mobility of subject	• Usually ambulant non-housebound • Usually ambulant housebound • Chairfast permanently • Bedfast permanently
**IADL disability**	
• Are you able to do the heavy housework? (IADL) • Are you able to shop and carry heavy bags? (IADL)	• No, needs help • Yes with some difficulty • Yes with no difficulty

Individuals who were missing information on functional status at the last interview and whose last available interview reported them to be disabled were classed as disabled on the assumption that functional disability is unlikely to improve.

#### Retrospective informant interviews (RINI)

In the period following brain donation, relatives were approached to ask whether they would be willing to give information about the deceased in the period prior to last illness or death. The aim of these retrospective informant interviews (RINI) was to gather details of any incident conditions or deterioration relevant to the final diagnosis formulated after brain examination. Informant-reported ADL and IADL were also collected. This information was used to calculate a disability status of all individuals at death.

There was insufficient information to assign functional status to seven non-demented individuals included in the sample; for these missing values no assumptions were made about the likely status and they were excluded from the analysis.

#### Dementia status

Dementia status (demented, non-demented or unknown) for each individual was assigned by a hierarchical system depending on the information available from each donor. Death certificates were checked to identify new dementia cases among those who were not demented in the last interview. In cases where this last interview was more than six months before death, the information from the RINI was used to clarify the dementia status of the individual at the time of death. When insufficient information was available from any of these sources, individuals were assigned a dementia status of "unknown"; this was the case in 8% of the autopsy sample (28/355). The majority of these individuals underwent just one interview only or their last interview was too long before death for dementia status to be assessed. These individuals were excluded from the analysis.

### Statistical methods

**Figure 1:**
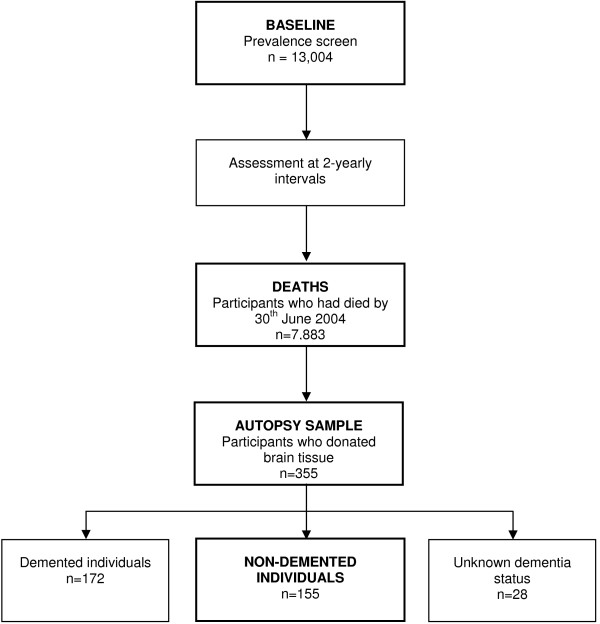
**The flow of individuals from recruitment until death and subsequent autopsy and examination of neuropathology**. Information on the number of participants who were approached to donate brain tissue but declined was not available at the time of writing.

MRC-CFAS has released data at defined points. For this study version 8.1 of the main dataset was used for the analysis of baseline characteristics of the initial screening cohort. The neuropathology dataset used was version 3.2. To allow a lag between death and notification, the analyses presented in this report are restricted to deaths before 30^th ^June 2004. All statistical analysis was conducted using Stata Software version 9.0 (Stata Corporation, College Station, Texas, USA). Comparisons between groups were performed using Pearson chi-square (χ^2^) analysis or Mann Whitney U tests as appropriate. All p-values are two sided. To model the effect of individual pathological variables on functional disability, odds ratios and their 95% confidence intervals were calculated using unconditional multinomial logistic regression. The analyses are based on non-demented individuals who participated in at least one interview of MRC-CFAS, and subsequently donated brain tissue. An overview of the flow of these individuals from recruitment to death and subsequent autopsy and analysis is presented in figure 1.

### Ethical approval

MRC CFAS has had ethical approval from Eastern Anglia Multicentre research ethics committee and all local ethical committees for the duration of the study (1990 to date). All individuals gave written informed consent.

## Results

Demographic characteristics (age, sex, education, social class) and functional disability status were compared between those who were alive at the censoring date (n = 5,166), all individuals who died (n = 7,883) and individuals who donated their brains (n = 355). Individuals who were alive were more likely to be female, from a higher social class, more highly educated and younger at the baseline interview (all p < 0.001). Individuals who donated brain tissue were slightly older at death, median 86 years versus 84 years for all deaths (p < 0.001), though there were no large differences in education, sex or social class, there was slight increase in individuals of non-manual social class within the donation sample.

A total of 355 individuals from Cambridge, Gwynedd, Newcastle, Nottingham and Oxford had donated brain tissue by 30^th ^June 2004. Of these, 172 (50%) were classified as demented before death, 155 (44%) as non-demented and for 28 (8%) the dementia status was unknown. Age at death in the 155 non-demented individuals ranged from 68 to 102 years, and the median age at death was 82 years for men and 86 for women. There were similar numbers of men and women (78 men and 77 women). One man and five women were aged 95 and over at death. The number of women steadily increased with each age group such that over half of the women in the sample were aged over 84 years. In contrast the men were more evenly distributed across the entire age range.

The median time from last assessment until death in non-demented individuals was 1.2 years (range 1 month – 7.3 years; 83% within 2 years of death). There were 84 RINI (22 in the 31 individuals with an interview more than 2 years from death). Hence, there are just nine individuals (6%) for whom the most recent information is more than two years old at time of death.

### Subcortical pathology

In the subcortical regions examined the most common pathological findings were NFT (61%; 94/154) and neuronal loss (46%; 71/154); plaques were present in 10% (14/137) of individuals. Moderate of severe pathology was seen for 22% of individuals who had neuronal loss, 28% who had plaques, 39% of the tangles and 55% of individuals with Lewy bodies. All individuals show these plaques in the nucleus basalis and one additionally showed plaques in the raphé. Lewy bodies in the SN were found in only seven individuals, with a further two individuals having Lewy bodies in the other areas examined. However it is likely that LB pathology was underestimated in the absence of α-synuclein immunocytochemistry. There was no difference between men and women in pathological burden. The frequency of all pathology subtypes increased with age with increases in plaques and NFT significant after adjusting for gender (p = 0.04 in each).

The overall prevalence of functional disability was 27% (42/153) for mild disability and 44% (68/153) for more severe disability, for two individuals disability status could not be ascertained. Disability was slightly more common in women than men, though the difference was not statistically significant. Stratification by age group showed that both mild and severe disability measures increased with age (p = 0.06 & p = 0.002 respectively), with all individuals over 95 years reporting functional disability (table 2). The risk of disability increased dramatically across age groups with individuals over 85 years having at least three times the risk of individuals in the youngest age group (table 3). Of the pathological variables Lewy bodies in any subcortical region, and in the SN, were predictors of mild disability, though they were less strongly associated with more severe disability. Neuronal loss appeared to be a significant predictor of severe functional disability, so that individuals who had mild-moderate neuronal loss had three times the risk of those with no loss of neurons (p = 0.008). Since all seven individuals with Lewy bodies in the SN had mild or severe functional disability, it was impossible to calculate an odds ratio. In this instance, Lewy bodies perfectly predicted an outcome of functional disability. Vascular effects in the subcortical region were rare (only 8 individuals), and hence risk cannot be accurately ascertained.

**Table 2:** Demographic characteristics and subcortical pathology of the non-demented individuals by disability status. n(%)

	**No disability**		**Mild disability**		**Severe disability**	
Total	43		42		68	
Age at death (years)						
<75	12	(28)	7	(17)	12	(18)
75–84	21	(49)	16	(38)	14	(21)
85+	10	(23)	19	(45)	42	(62)
Men	27	(63)	18	(43)	32	(46)
Women	16	(37)	24	(57)	36	(55)
NFT	22	(51)	28	(68)	43	(63)
Neuronal loss	14	(33)	16	(39)	40	(59)
Plaques	6	(16)	4	(10)	3	(5)
Vascular disease	1	(2)	2	(5)	5	(8)
Lewy bodies	1	(2)	5	(12)	3	(4)
Lewy bodies in SN only	0	(0)	4	(10)	3	(4)

**Table 3:** The association of functional disability with subcortical measure of neurodegeneration.

	**Unadjusted Odds Ratio (95% CI)**				**Adjusted Odds Ratio (95% CI)**^†^			
	Mild		Severe		Mild		Severe	

**Sex**								
Men	1.00		1.00		1.00		1.00	
Women	2.3	(0.9–5.4)	1.9	(0.9–4.1)	3.4	(0.9–12.9)	1.4	(0.5–4.2)
**Age at death**								
<75	1.00		1.00		1.00		1.00	
75–84	1.3	(0.4–4.1)	0.7	(0.2–1.9)	1.2	(0.2–7.0)	0.3	(0.1–1.4)
>84	3.3	(1.0–10.9)	4.2	(1.5–12.1)	2.9	(0.5–16.2)	2.4	(1.0–9.2)
Overall effect*	1.7	(1.0–3.0)	2.3	(1.4–3.8)	1.6	(0.7–3.5)	1.7	(0.9–3.2)

**SUBCORTICAL PATHOLOGY ***(Factor present reference absence)*								

**NFT**	2.1	(0.8–5.0)	1.6	(0.8–3.6)				
**Neuronal loss**	1.3	(0.5–3.2)	3.0	(1.3–6.6)	1.4	(0.3–5.4)	3.9	(1.2–12.5)
**Plaques**	0.6	(0.1–2.2)	0.3	(0.1–1.2)				
**Vascular disease**	2.2	(0.2–24.9)	3.3	(0.4–29.1)				
**Lewy bodies**	8.3	(0.9–78.3)	1.8	(0.2–18.6)	8.1	(0.8–87.2)	1.2	(0.1–14.2)
***LB in Substantia nigra***		*Impossible to calculate; 100% predictive*						

*χ^2 ^test for trend ^† ^Adjusted for all other effects in the table.

In assessing the combined effect of neuropathological variables on functional disability, the multivariable model showed that advanced age (>84 years) and neuronal loss showed increased severe functional disability independently of each other, though other factors were still at increased risk.

Analyses were performed to assess how robust the results were to uncertain decisions or assumptions that were made about the data. Excluding 28 individuals with reported stroke from the analysis, on the basis that stroke is an established cause of functional disability, had no effect on the odds ratios derived from the multivariate model, thus confirming that functional disability following stroke did not distort the observed clinicopathological relationship between function and neuronal loss. Excluding information from RINI or those who died more than one year from last interview had no effect on the odds ratios.

## Discussion

In this population-based group of donors who were not demented before death we found that pathological measures in subcortical areas were associated with functional disability. Plaques were not associated with impairment and estimation of the risk for impairment associated with NFT was not significant but greater than one. Lewy bodies in any area were associated with greater levels of disability. Those individuals with Lewy bodies in the substantia nigra were all disabled. Neuronal loss was associated with severe disability, even after adjustment for Lewy bodies, indicating these two factors are independently associated with disability. Factors leading to neuronal loss in the absence of a definable pathology like Lewy bodies therefore emerge as a relevant but poorly characterised issue in clinicopathological correlative studies. For the present study the lack of data on neuritic synucleinopathy is a significant limitation.

The study shows the high frequency of functional disability in non-demented individuals aged 65 years and older in England and Wales, and highlights the possible role of neuronal loss in subcortical regions as a potential substrate for functional decline. All individuals with Lewy bodies in the SN were found to be disabled. A recent study of 86 older persons revealed no relation between nigral Lewy bodies and gait impairment typical of Parkinson's disease after controlling for dementia status but, contrary to the findings of this study, did find an association between NFTs and functional impairment in non-demented individuals[27].

The study has a number of drawbacks. Those who are impaired are more likely to drop out, thus those individuals who continue are possibly less disabled than those who do not, but this is balanced by the fact that many individuals develop disability in the period leading up to death. The donor population are slightly older and with more functional impairment than the rest of the population that had died; this is partly due to the sampling strategy which is biased towards those who are cognitively frail. The demented donors were excluded from the analyses presented here. Loss from the population who made a 'Declaration of Intent' to donate their brains to the actual donors may be associated with increasing frailty but this is unlikely to lead to bias in the relationship being examined here. The measures of disability are taken from self- and informant-reports although it has been reported that self-reported disability is reasonably accurate[28,29]. The measures of disability may not be sensitive enough to brain changes which may require the use of objective measurement. The time interval between measurement and death may affect relationships but these are likely to attenuate measured associations. Though there is some possibility that in a minority of individuals function may improve. The pathological data available for this analysis was not specifically selected for a study of functional ability. The CERAD protocol was designed to capture data relevant to dementia status not physical function. It is biased towards capturing critical thresholds of severity in cortical not subcortical structures. Lewy bodies, a key neuropathological variable, were detected using methods (H&E staining and ubiquitin immunocytochemistry) that precede the development of methods based on α-synuclein. Whilst the morphology of Lewy bodies in all these five brain regions is readily demonstrated by H&E it is recognised that additional Lewy-related pathology (e.g. Lewy neurites) would not be detected. Thus the relationships shown in this paper merit more detailed investigation.

The study is the largest to address the association between neuropathological indices in subcortical areas and functional disability in a non-demented cohort, However the numbers who exhibit neuropathological changes such as Lewy bodies are still small. The difference seen in the association between Lewy bodies and the severity of disability could be due to numerical differences. The causes of functional disability are often perceived to be musculoskeletal and peripheral nervous system problems (which can also arise from synucleinopathy) but this study and the small number of other similar studies suggest that a more complex interpretation, integrating CNS changes, may be appropriate[17,18].

In comparison with previous findings, the reported association between neuronal loss and functional disability supports evidence that neuronal loss may be a feature of brain ageing[21,27,30]. Studies arguing that a decline in neuronal number is not significantly associated with normal ageing[31,32] have not focussed on subcortical populations. Given the heterogeneity in regional vulnerability demonstrable in other age-related cerebral pathologies, it remains possible that neuronal loss associated with functional disability can occur through ageing. It is possible that the observed relationship between neuronal loss and function may be causal. However given that the NbM, raphé nuclei, locus ceruleus or DEX are not conventionally associated with motor function suggests that the results may reflect co-morbidity. However brain regions conventionally regarded as 'motor', such as the cerebellum, are increasingly invoked in relation to additional non-motor functions An alternative explanation is that neuronal loss may occur as a result of other age-related changes responsible for the loss in function, i.e. neuronal loss could have been a pathological confounder. The most likely co-morbid confounder is cerebrovascular disease, so that we looked at the potential impact of pathological confounding by repeating the analysis to exclude patients in whom examination of the brain showed evidence of a stroke since these individuals have vascular pathology likely to cause functional decline. The analysis showed no obvious confounding but the reanalysis does not exclude an effect of cerebrovascular disease in the absence of clinical stroke or cerebral infarction. Including vascular changes within the subcortical regions themselves did not provide more insight, though these vascular changes were rare in this cohort. This question requires further examination in a larger sample with greater statistical power. Similarly, the role of Lewy bodies in the SN and functional disability warrants further research on a larger sample size. Given that the findings are focused on selected areas of the brain, additional investigations in the context of the brain as a whole may clarify the issue of pathological confounding.

## Conclusion

The data presented offer new insights into the relationship between changes in the brain and functional ability in older people who are cognitively intact. Whilst the limitations of the study preclude the establishment of clear causal mechanisms, the findings indicate a neglected area of clinicopathological enquiry into ageing and the brain.

## Competing interests

The authors declare that they have no competing interests.

## Authors' contributions

MB Undertook the first analysis, wrote the first draft of the paper. CB, IM, PI provided expert clinical input, guarantors of the study integrity and edited the paper. MC Supervised the initial investigate and edited the paper. FM Undertook the final analyisis, edited the final draft of the paper and is guarantor on the analyisis. MRC CFAS neuropathology group is the study collaborators and provides the data integrity, research governance and comments on pre-submission drafts of the paper. All named authors read and approved the final manuscript.

## Pre-publication history

The pre-publication history for this paper can be accessed here:


